# Chimpanzee Personality and the Arginine Vasopressin Receptor 1A Genotype

**DOI:** 10.1007/s10519-016-9822-2

**Published:** 2016-11-02

**Authors:** V. A. D. Wilson, A. Weiss, T. Humle, N. Morimura, T. Udono, G. Idani, T. Matsuzawa, S. Hirata, M. Inoue-Murayama

**Affiliations:** 1 Department of Psychology, School of Philosophy, Psychology and Language Sciences, The University of Edinburgh, 7 George Square, Edinburgh, EH8 9JZ UK; 2 Cognitive Ethology, German Primate Center, Georg-August-University Goettingen, Leibniz-ScienceCampus, Göttingen, Germany; 3 Durrell Institute of Conservation and Ecology (DICE), School of Anthropology and Conservation, University of Kent, Canterbury, UK; 4 Wildlife Research Center, Kyoto University, Kyoto, Japan; 5 Institute for Advanced Study, Kyoto University, Kyoto, Japan; 6 Primate Research Institute, Kyoto University, Inuyama, Japan; 7 Japan Monkey Centre, Inuyama, Japan; 8 National Institute for Environmental Studies, Tsukuba, Japan

**Keywords:** Animal model, AVPR1a, Chimpanzee, Heritability, Personality, Vasopressin

## Abstract

Polymorphisms of the arginine vasopressin receptor 1a (AVPR1a) gene have been linked to various measures related to human social behavior, including sibling conflict and agreeableness. In chimpanzees, AVPR1a polymorphisms have been associated with traits important for social interactions, including sociability, joint attention, dominance, conscientiousness, and hierarchical personality dimensions named low alpha/stability, disinhibition, and negative emotionality/low dominance. We examined associations between AVPR1a and six personality domains and hierarchical personality dimensions in 129 chimpanzees (*Pan troglodytes*) living in Japan or in a sanctuary in Guinea. We fit three linear and three animal models. The first model included genotype, the second included sex and genotype, and the third included genotype, sex, and sex × genotype. All personality phenotypes were heritable. Chimpanzees possessing the long form of the allele were higher in conscientiousness, but only in models that did not include the other predictors; however, additional analyses suggested that this may have been a consequence of study design. In animal models that included sex and sex × genotype, chimpanzees homozygous for the short form of the allele were higher in extraversion. Taken with the findings of previous studies of chimpanzees and humans, the findings related to conscientiousness suggest that AVPR1a may be related to lower levels of impulsive aggression. The direction of the association between AVPR1a genotype and extraversion ran counter to what one would expect if AVPR1a was related to social behaviors. These results help us further understand the genetic basis of personality in chimpanzees.

**Electronic supplementary material:**

The online version of this article (doi:10.1007/s10519-016-9822-2) contains supplementary material, which is available to authorized users.

## Introduction

Arginine vasopressin is a neuropeptide involved in the regulation of the hypothalamic–pituitary–adrenal axis and implicated in species differences in affiliative and aggressive behaviors (Bielsky et al. 2004). Vasopressin has three receptor types. Two (AVPR1a and AVPR1b) have been implicated in social behavior, although the majority of this work has been focused on AVPR1a (Bielsky et al. 2004; Caldwell et al. 2008; Wersinger et al. 2002). In prairie voles (*Microtus ochrogaster*), a species with strong partner preferences, a repeat sequence of a microsatellite region in the 5′ flanking region of AVPR1a gene is present; this repeat sequence is shorter in montane voles (*M. montanus*), a closely-related species which does not form strong partner preferences (Nair and Young 2006). However, further analyses of AVPR1a across 21 *Microtus* species did not find an association between partner preferences and the AVPR1a genotype (Fink et al. 2006).

Recent research into cooperative breeding African cichlids found species-specific differences in arginine vasotocin expression relative to prosocial behavior; when social species were compared with non-social species, brain expression of vasotocin was higher for some social versus non-social species, but this pattern was not consistent (O’Connor et al. 2015). Similarly, a study of AVPR1a polymorphisms across three species of Old World monkeys (family Cercopithecidae), three species of gibbon (family Hylobatidae), and five great ape species (family Hominidae) found no association between the receptor polymorphism and mating behavior (Rosso et al. 2008). The authors did note however that they did not examine brain distributions of AVPR1a receptors in relation to species-specific behavior, an important consideration for understanding links between genotype, neurobiology and behavior (Rosso et al. 2008). The evidence that vasopressin and its homologs mediate species differences in vertebrate social behavior is therefore mixed.

Researchers have also examined within-species associations between vasopressin and behavior. Early work assessed the association between vasopressin and scent marking in Syrian hamsters (*Mesocricetus auratus*). Scent marking in Syrian hamsters is higher in high ranking individuals, and vasopressin injections made into the medial preoptic area of the hypothalamus led to increases in scent marking (Ferris et al. 1984). Later studies of Syrian hamsters found that orally administered AVPR1a antagonists inhibit male aggression (Ferris et al. 2006). Similar results have recently been found in cooperatively breeding cichlids (*Neolamprologus pulcher*), with brain expression of vasotocin being higher in subordinate individuals (Reddon et al. 2015).

Studies also focused on the role of vasopressin in modulating behavioral pathways in humans. For example, intranasal administrations of vasopressin produced an increase in salivary cortisol during social stress (Ebstein et al. 2009), and were associated with reciprocity of cooperation in men (Rilling et al. 2012), and enhanced encoding of emotionally valenced facial expressions (Guastella et al. 2010). The role of vasopressin in emotion processing has further been linked to changes in prefrontal cortex and amygdala activation during a facial expression matching task (Zink et al. 2010).

Findings in humans, as well as those showing links between AVPR1a polymorphisms, vasopressin, and social behavior in nonhuman animals (e.g., Nair and Young 2006) encouraged researchers to carry out candidate gene studies of AVPR1a polymorphisms in humans. In humans, the RS3 microsatellite occurs within the Dup B region of the vasopressin receptor gene (Thibonnier et al. 2000), and is accompanied by the Dup A region (Donaldson et al. 2008), as in other great apes (Donaldson et al. 2008; Hammock and Young 2005). Several studies reported links between AVPR1a genotype and human behavior and personality. For example, men who are carriers of the RS3 334 bp allele of AVPR1a scored lower on a scale that assessed affiliation towards and time spent with their partner (Walum et al. 2008). The RS3 region has also been linked to traits of social appropriateness and sibling conflict (Bachner-Melman et al. 2005), and long forms of the RS3 region (i.e. 327–343 bp) have been associated with higher levels of AVPR1a mRNA in the hippocampus (Knafo et al. 2008). Of the few studies that examined links between AVPR1a and personality, one found an association between a non-synonymous SNP located on the vasopressin gene and higher agreeableness; however, this effect did not survive correction for multiple tests (Haram et al. 2014). Additionally, a gene enrichment analysis of candidate genes for aggression found an association between AVPR1a and aggression in nearly 19,000 children (Pappa et al. 2016).

Recent studies examined the role of AVPR1a polymorphisms in the behavior and personality of chimpanzees (*Pan troglodytes*). Unlike humans and other great apes, chimpanzees are polymorphic for the deletion of the Dup B site, including the RS3 microsatellite (Donaldson et al. 2008; Hammock and Young 2005). Hopkins et al. (2012) and Latzman et al. (2014) examined associations between polymorphisms of the Dup B region of AVPR1a and personality in 83 and 116 chimpanzees, respectively. The personality domains used in Hopkins et al. (2012) were based on a four component structure that was found in chimpanzees at the Yerkes National Primate Center and chimpanzees housed in US and Australian zoological parks (see Weiss et al. 2007 for details). The personality variables used in Latzman et al. (2014) represented hierarchical personality dimensions derived using a two-step procedure (see Goldberg 2006 for details). In the first step principal components analyses were used to extract and obtain component scores for two, three, four, five, and six component solutions. In the second step correlations between component scores that represented associations between components at higher and lower levels of the hierarchy, i.e., between component scores from structures with fewer and more dimensions, respectively, were computed. In these studies, Hopkins et al. (2012) and Latzman et al. (2014) did not find significant main effects of genotype, but they did find significant sex × by genotype interactions. Specifically, Hopkins et al. (2012) found that, among chimpanzees who possessed the long form of the Dup B allele, males scored higher than females on the dominance domain and lower than females on the conscientiousness domain. Similarly, Latzman et al. (2014) found that the male advantage in the hierarchical personality dimensions “(low) alpha/stability” and “disinhibition” at the levels of the two- and three-component levels, respectively, was greater among chimpanzees who possessed the long form of the Dup B allele. Latzman et al. also found that the female advantage in a hierarchical personality dimension at the three-component level, “negative emotionality/low dominance,” was greater among chimpanzees who possessed the long form of the Dup B allele.

Three further studies of this AVPR1a polymorphism in chimpanzees demonstrate its association with traits related to social behavior. Hopkins et al. (2014) found significant sex, genotype, and the sex × genotype interaction effects on performance in a receptive joint attention task: males with the long form of the Dup B allele demonstrated better performance than males who were homozygous for the deletion. Anestis et al. (2014) found that chimpanzees with a copy of the *L* allele (lacking the RS3 deletion) had higher scores on “smart” (“Uses coalitions”, “Gets groomed frequently”, “Has play offers accepted”) and in males, higher scores on “friendly” (“Directs affiliative behaviors to all group members”). Finally, Staes et al. (2015) reported that male chimpanzees homozygous for the long allele, and female heterozygotes, groomed and were groomed by others more frequently.

Building on these findings, and especially the work of Hopkins et al. (2012) and Latzman et al. (2014), we tested whether the long form of the AVPR1a genotype was associated with any of the six chimpanzee personality domains—dominance, extraversion, conscientiousness, agreeableness, neuroticism, and openness—identified in an earlier study (King and Figueredo 1997) or the hierarchical personality dimensions of (low) alpha/stability, disinhibition, and negative emotionality/low Dominance, that were related to genotype in Latzman et al. (2014). Because these and other studies found evidence for sex × genotype interactions, we also tested for this interaction.

Our study differed in two ways from the work of Hopkins et al. (2012) and Latzman et al. (2014). Firstly, we used a more recent version of the personality questionnaire than did the studies of Hopkins et al. (2012) and Latzman et al. (2014). Our questionnaire thus included 11 additional items (see Weiss et al. 2009 for details). Furthermore, unlike Hopkins et al. (2012) we tested for associations between genotype and all six personality domains, and not just the dominance, extraversion, conscientiousness, and agreeableness domains, which generalized from chimpanzees living in zoos in the United States and Australia to chimpanzees living in Yerkes National Primate Center (Weiss et al. 2007).

The second difference concerns our analytic approach. Hopkins et al. (2012) and Latzman et al. (2014) tested for associations between personality constructs and genotypes by means of linear models (multivariate analyses of covariance followed by univariate analyses of covariance). In both cases, to adjust for relatedness, the models included a covariate that indicated, for each chimpanzee, his or her relatedness with all other chimpanzees in their pedigree. Furthermore, Latzman et al. (2014) but not Hopkins et al. (2012) tested for rearing history effects and the two- and three-way interactions between sex, rearing history, and genotype. For our study we also fit linear models, but we did not include rearing effects because Latzman et al. (2014) did not find any significant main effects of rearing or interactions of rearing with sex or genotype. In addition, unlike Latzman et al. (2014) and Hopkins et al. (2012), but similar to Hopkins et al. (2014), we controlled for relatedness by fitting ‘animal models’. The animal model is a type of mixed effects model in which the degree to which subjects deviate from the mean on some trait, i.e., the random effects of individuals, are not treated as independent, but as being more similar between genetically related individuals (Kruuk 2004). The animal model accomplishes this by using a matrix that describes the genetic relatedness (Wright’s coefficient of relatedness) between all pairs of individuals to estimate how much each individual deviates from the trait’s mean (Kruuk 2004). As such, when fixed effects, such as genotype, are included in an animal model, the relatedness among all pairs of individuals is taken into account. In other words, these models can estimate the effects of a candidate gene on a phenotype while controlling for the tendency for related individuals to resemble one another more closely on that phenotype and to be more likely to share the candidate gene (Kruuk 2004). Animal models therefore eliminate the possibility that the phenotype and gene are inherited together but are not causally related and thus provide a robust method for assessing personality-genotype relationships in samples of related individuals (Kruuk 2004). One further benefit of animal models is that, because they provide an estimate of the additive genetic variance underlying the phenotype under study, they provide heritability estimates. Given the small number of studies on the heritability of personality in chimpanzees (Latzman et al. 2015; Weiss et al. 2000), obtaining heritability estimates of chimpanzee personality in another sample will be valuable.

## Methods

### Subjects

Subjects were drawn from chimpanzees in zoological parks, research centers, and a sanctuary, all located in Japan (*N* = 124), or in a sanctuary in Guinea (*N* = 19). To avoid stratification, we excluded chimpanzees whose subspecies was not *Pan troglodytes verus* (1 *P. t. schweinfurthii*, 1 *P. t. troglodytes*, 10 hybrids, and 2 unknown). The remaining 129 chimpanzees (69 females and 60 males) included 110 chimpanzees who lived in 11 facilities in Japan and the 19 wild chimpanzees in Guinea. The ages of the chimpanzees ranged from 1.7 to 51.7 (mean ± SD = 20.5 ± 10.7).

### Genotypes

DNA was extracted from blood or fecal samples (Hong et al. 2009). Genotyping of the AVPR1a DupB region was conducted following Latzman et al. (2014). We used a forward primer 5′-GCATGGTAGCCTCTCTTTAAT-3′ and a reverse primer 5′-CATACACATGGAAAGCACCTAA-3′ (synthesized following Donaldson et al. 2008) and *LA*-*Taq* DNA polymerase (TaKaRa, Shiga, Japan) for PCR amplification with an annealing temperature of 55 °C for 35–40 cycles. PCR products were resolved on a 2 % agarose gel. The DupB-containing allele (*L*) resulted in a band of 900 base pairs, while the DupB minus allele (*S*) was 570 base pairs long. Genotyping was repeated at least twice to check the result.

A total of 145 chimpanzees were initially genotyped. The genotypes of two chimpanzees were uncertain. We excluded these individuals. Of the 129 successfully genotyped chimpanzees who were *P. t. verus*, 94 were homozygous for the S allele (*SS*), 5 were homozygous for the L allele (*LL*), and 30 were heterozygous (*LS*). Because of the low number of *LL* chimpanzees, we conducted an exact test (Graffelman and Morales-Camarena 2008) using the HardyWeinberg package (Graffelman 2015) in R (R Core Team 2015) to test whether these genotypes were in Hardy–Weinberg equilibrium. The SELOME p value (0.19) indicated that these genotypes were in Hardy–Weinberg equilibrium. The less conservative mid p value (0.13) arrived at the same conclusion.

### Personality ratings

Chimpanzees were rated on the Hominoid Personality Questionnaire (HPQ; Weiss et al. 2009), an expanded version of the Chimpanzee Personality Questionnaire and the Orangutan Personality Questionnaire (for details see King and Figueredo 1997; Weiss et al. 2006). The Chimpanzee Personality Questionnaire was used in the previous studies on AVPR1a genotype and personality (Hopkins et al. 2012; Latzman et al. 2014).

The HPQ consists of 54 trait descriptive adjectives followed by one to three sentences that set the adjective in the context of primate behavior. For example, the item ‘fearful’ reads “**FEARFUL**: Subject reacts excessively to real or imagined threats by displaying behaviors such as screaming, grimacing, running away or other signs of anxiety or distress.” The questionnaire instructs raters to use a 7-point scale to rate chimpanzees on the item where “1” is defined as “displays either total absence or negligible amounts of the trait” and “7” is defined as “displays extremely large amounts of the trait.” Raters were also instructed not to discuss their ratings.

The chimpanzees in Japan and in Guinea were rated on a Japanese and a French translation of the HPQ, respectively. The psychometric properties of the Japanese translation were comparable to the English language version of the Chimpanzee Personality Questionnaire (Weiss et al. 2009). The psychometric properties of a French translation of the Chimpanzee Personality Questionnaire, which was not used in this study, were comparable to the English language version of the same questionnaire (King et al. 2005).

### Analyses

#### Variables

The dependent variables were scores representing the six chimpanzee personality domains described by Weiss et al. (2009) and three hierarchical personality dimensions—low alpha, disinhibition, and negative emotionality/low dominance—described by Latzman et al. (2014). To create the dependent personality variables, we first obtained mean item scores across raters. We then used these scores to create unit-weighted scores for each domain or dimension (see ESM Table S1). For ease of interpretation, we transformed these variables into *z*-scores (mean ± SD = 0 ± 1). The independent variables included sex (female = 0, male = 1) and genotype (*L* carriers = 0, *SS* = 1).

#### Modeling

Statistical analyses were conducted using R (R Core Team 2015). To test whether personality domains were associated with the AVPR1a genotype we first fit three linear models for each personality variable using the lm function (R Core Team 2015). The first linear model included genotype as the sole effect, the second linear model included the effects of sex and genotype, and the third linear model included the effects of sex, genotype, and sex × genotype.

We then fit three animal models for each personality variable using the MCMCglmm function (Hadfield 2010). These models were identical to the linear models in that the first included the fixed effect of genotype as the sole effect, the second included the fixed effects of sex and genotype, and the third included the fixed effects of sex, genotype, and the sex × genotype interaction. All three animal models also included subject identity as a random effects term that was conditioned on the relatedness matrix, which was generated by MCMCglmm from our chimpanzee pedigree. The paternity and maternity data in this pedigree for the 129 chimpanzees housed in Japan were obtained from the Great Ape Information Network (http://www.shigen.nig.ac.jp/gain/index.jsp). The sire and dam were known for 68 subjects, providing pedigree data for 90 chimpanzees. Sire and dam were unknown for all 19 chimpanzees in Guinea. To estimate fixed and random effects, MCMCglmm uses Markov Chain Monte Carlo estimation to determine the parameters of a posterior distribution and uses an inverse-Gamma distribution as the prior for variance components (Hadfield 2010). We specified priors with a belief parameter (ν) of 0.75 and a covariance matrix (**V**) of 0.5. We ran the models for 10,000,000 iterations, had a burn in period of 6,000,000, and thinned the samples from the posterior distribution to 1000.

## Results

### Linear models

**Fig. 1:**
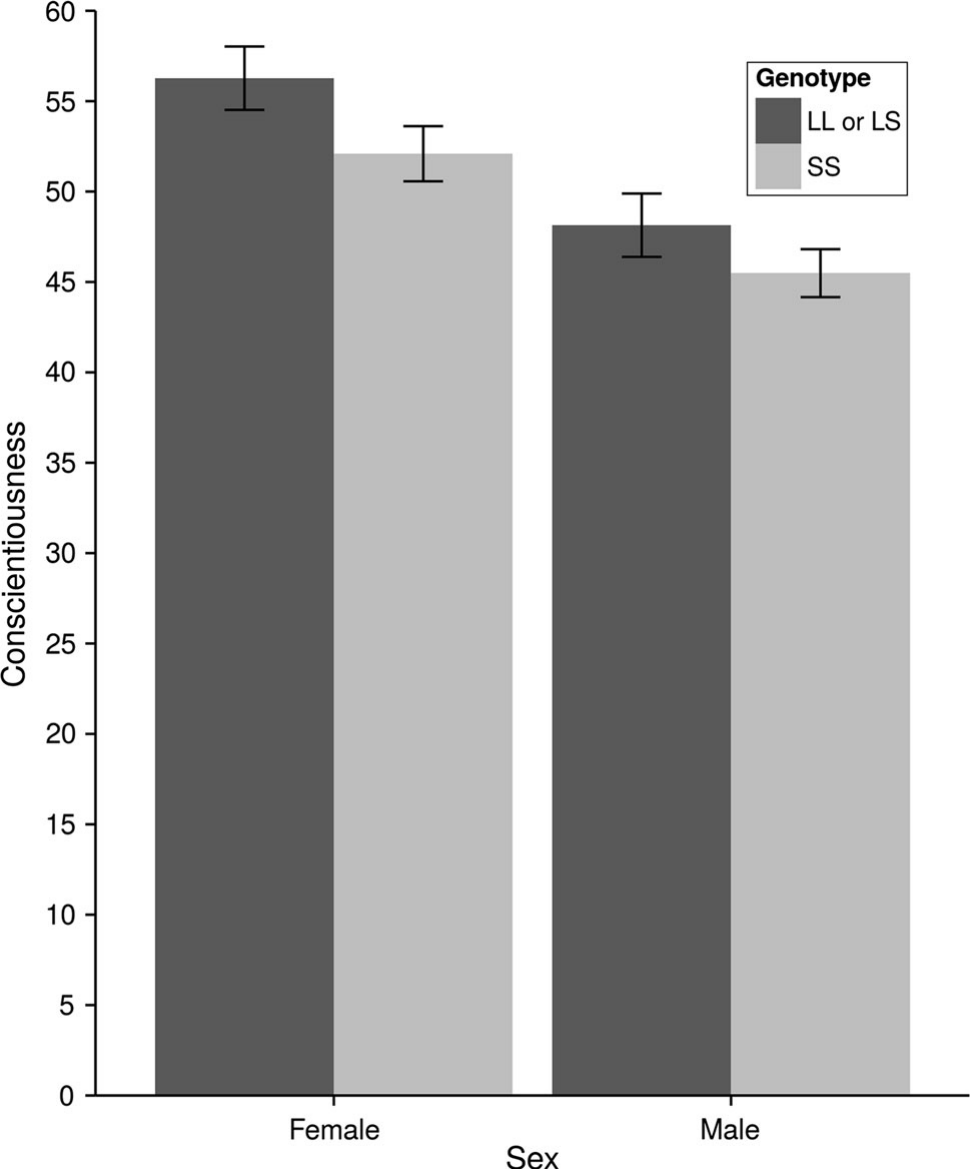
Mean conscientiousness scores in *T*-score units (mean ± SD = 50 ± 10) for males and females by AVPR1a genotype. *Error bars* represent standard errors

The results for the linear models are presented in Table 1. There were significant sex effects. In models that only adjusted for sex, males were higher in dominance, lower in conscientiousness, higher in low alpha and disinhibition, and lower in negative emotionality/low dominance. In the fully adjusted models, males were higher in extraversion, lower in conscientiousness, higher in low alpha, and higher in disinhibition. There was only one significant effect of genotype: in the unadjusted model, subjects who were homozygous for the *S* allele were lower in conscientiousness than those who were *L* allele carriers (see Fig. 1). None of the sex × genotype interaction effects were significant.

**Table 1:** Linear model results for the effects of AVPR1 genotype, sex, and sex × genotype on personality domains and hierarchical personality dimensions

	Unadjusted				Sex adjusted				Fully adjusted			
	b	SE	l-95 %	u-95 %	b	SE	l-95 %	u-95 %	b	SE	l-95 %	u-95 %
Dominance												
Intercept	−0.12	0.17	−0.46	0.21	−0.30	0.18	−0.65	0.05	−0.36	0.21	−0.77	0.05
*SS* vs. *LL* + *LS*	0.17	0.20	−0.23	0.56	0.10	0.19	−0.28	0.49	0.19	0.25	−0.31	0.69
Sex	–	–	–	–	**0.49**	**0.17**	**0.14**	**0.83**	0.64	0.34	−0.04	1.32
*SS* vs. *LL* + *LS* × sex	–	–	–	–	–	–	–	–	−0.21	0.40	−0.99	0.58
Extraversion												
Intercept	−0.14	0.17	−0.48	0.19	−0.22	0.18	−0.58	0.14	−0.44	0.21	−0.85	−0.02
*SS* vs. *LL* + *LS*	0.20	0.20	−0.19	0.59	0.17	0.20	−0.22	0.57	0.49	0.26	−0.01	1.00
Sex	–	–	–	–	0.20	0.18	−0.15	0.55	**0.78**	**0.35**	**0.10**	**1.47**
*SS* vs. *LL* + *LS* × sex	–	–	–	–	–	–	–	–	−0.79	0.40	−1.59	0.00
Conscientiousness												
Intercept	0.33	0.17	−0.00	0.65	0.59	0.17	0.25	0.92	0.63	0.20	0.24	1.02
*SS* vs. *LL* + *LS*	**−0.45**	**0.19**	**−0.83**	**−0.06**	−0.36	0.18	−0.72	0.01	−0.42	0.24	−0.89	0.06
Sex	–	–	–	–	**−0.70**	**0.16**	**−1.02**	**−0.38**	**−0.81**	**0.32**	**−1.45**	**−0.17**
*SS* vs. *LL* + *LS* × sex	–	–	–	–	–	–	–	–	0.15	0.38	−0.59	0.90
Agreeableness												
Intercept	−0.16	0.17	−0.49	0.18	−0.16	0.18	−0.52	0.20	−0.18	0.21	−0.60	0.25
*SS* vs. *LL* + *LS*	0.22	0.20	−0.18	0.61	0.21	0.20	−0.18	0.61	0.23	0.26	−0.28	0.75
Sex	–	–	–	–	0.02	0.18	−0.34	0.37	0.05	0.35	−0.65	0.75
*SS* vs. *LL* + *LS* × sex	–	–	–	–	–	–	–	–	−0.04	0.41	−0.85	0.77
Neuroticism												
Intercept	−0.13	0.17	−0.46	0.21	−0.13	0.18	−0.49	0.23	−0.24	0.21	−0.67	0.18
*SS* vs. *LL* + *LS*	0.17	0.20	−0.22	0.56	0.17	0.20	−0.23	0.57	0.34	0.26	−0.18	0.85
Sex	–	–	–	–	0.01	0.18	−0.34	0.37	0.32	0.35	−0.38	1.02
*SS* vs. *LL* + *LS* × sex	–	–	–	–	–	–	–	–	−0.41	0.41	−1.22	0.39
Openness												
Intercept	0.01	0.17	−0.33	0.35	0.05	0.18	−0.31	0.41	0.04	0.22	−0.39	0.46
*SS* vs. *LL* + *LS*	−0.01	0.20	−0.41	0.38	0.00	0.20	−0.40	0.40	0.02	0.26	−0.50	0.53
Sex	–	–	–	–	−0.10	0.18	−0.46	0.25	−0.07	0.35	−0.77	0.63
*SS* vs. *LL* + *LS* × sex	–	–	–	–	–	–	–	–	−0.04	0.41	−0.86	0.77
Low alpha												
Intercept	−0.19	0.17	−0.52	0.14	−0.41	0.17	−0.76	−0.07	−0.48	0.20	−0.88	−0.08
*SS* vs. *LL* + *LS*	0.26	0.20	−0.13	0.65	0.18	0.19	−0.19	0.56	0.28	0.25	−0.21	0.77
Sex	–	–	–	–	**0.60**	**0.17**	**0.27**	**0.94**	**0.78**	**0.33**	**0.12**	**1.44**
*SS* vs. *LL* + *LS* × sex	–	–	–	–	–	–	–	–	−0.24	0.39	−1.01	0.53
Disinhibition												
Intercept	−0.21	0.17	−0.54	0.12	−0.39	0.18	−0.74	−0.05	−0.48	0.21	−0.89	−0.07
*SS* vs. *LL* + *LS*	0.29	0.20	−0.10	0.68	0.23	0.19	−0.16	0.61	0.35	0.25	−0.15	0.84
Sex	–	–	–	–	**0.49**	**0.17**	**0.15**	**0.83**	**0.71**	**0.34**	**0.04**	**1.39**
*SS* vs. *LL* + *LS* × sex	–	–	–	–	–	–	–	–	−0.30	0.39	−1.08	0.48
Negative emotionality/low dominance												
Intercept	0.06	0.17	−0.28	0.39	0.24	0.18	−0.11	0.59	0.24	0.21	−0.17	0.66
*SS* vs. *LL* + *LS*	−0.08	0.20	−0.47	0.32	−0.01	0.19	−0.40	0.37	−0.02	0.25	−0.52	0.48
Sex	–	–	–	–	**−0.49**	**0.17**	**−0.83**	**−0.15**	−0.51	0.34	−1.19	0.17
*SS* vs. *LL* + *LS* × sex	–	–	–	–	–	–	–	–	0.02	0.40	−0.77	0.81

Personality domains and hierarchical personality dimensions were converted into z-scores for these analyses. l-95 % and u-95 % represent the lower and upper bounds of the 95 % confidence interval, respectively. Significant values highlighted in bold (p < 0.05)

### Animal models

The trace plots for the animal models did not suggest the presence of autocorrelations and the density plots indicated that the distributions around the estimates were approximately normal. Data used to create trace and density plots are available at https://github.com/alexweissuk/avpr1a-chimpanzee.git.

The personality domains and the hierarchical personality dimensions were heritable in all models. The heritabilities across models and phenotypes ranged from 0.13 to 0.44, the median heritability was 0.24, and none of the credible intervals included 0 (see Table 2).

**Table 2:** Heritability estimates for each personality domain and hierarchical personality dimensions

	Unadjusted			Sex adjusted			Fully adjusted		
	h^2^	l-95 %	u-95 %	h^2^	l-95 %	u-95 %	h^2^	l-95 %	u-95 %
Domains									
Dominance	0.24	0.04	0.44	0.21	0.04	0.41	0.21	0.04	0.41
Extraversion	0.44	0.10	0.76	0.42	0.10	0.75	0.41	0.10	0.74
Conscientiousness	0.19	0.03	0.41	0.21	0.04	0.44	0.21	0.03	0.44
Agreeableness	0.28	0.04	0.56	0.28	0.04	0.57	0.28	0.04	0.57
Neuroticism	0.14	0.03	0.28	0.14	0.03	0.28	0.13	0.03	0.27
Openness	0.25	0.04	0.52	0.27	0.04	0.57	0.27	0.04	0.57
Hierarchical dimensions									
Low alpha	0.27	0.04	0.55	0.27	0.06	0.56	0.27	0.05	0.55
Disinhibition	0.23	0.03	0.49	0.24	0.04	0.50	0.25	0.04	0.51
Negative emotionality/low dominance	0.17	0.04	0.35	0.16	0.03	0.31	0.16	0.03	0.32

l-95 % and u-95 % represent the lower and upper bounds of the 95 % credible interval, respectively

The results of the animal models are presented in Table 3. As in the linear models, there were significant sex effects. In sex adjusted models males were higher in dominance, lower in conscientiousness, and higher in low alpha and disinhibition. In fully adjusted models, males were higher in extraversion, lower in conscientiousness, and again, higher in low alpha and disinhibition. There were also two significant effects of genotype. First, in the unadjusted model subjects who were homozygous for the *S* allele were lower in conscientiousness than those who were *L* allele carriers. Second, in the fully adjusted model subjects who were homozygous for the *S* allele were higher in extraversion than those who were *L* allele carriers. As in our linear models, none of the sex × genotype interactions were significant.

**Table 3:** MCMCglmm results for the effects of AVPR1 genotype, sex, and sex × genotype on personality domains and hierarchical personality dimensions

	Unadjusted				Sex adjusted				Fully adjusted			
	b	l-95 %	u-95 %	N_eff_	b	l-95 %	u-95 %	N_eff_	b	l-95 %	u-95 %	N_eff_
Dominance												
Intercept	−0.10	−0.45	0.24	4000.00	−0.26	−0.62	0.11	4622.37	−0.32	−0.72	0.10	4000.00
*SS* vs. *LL* + *LS*	0.13	−0.26	0.54	4000.00	0.07	−0.33	0.46	4671.12	0.16	−0.28	0.69	3513.66
Sex	–	–	–	–	**0.44**	**0.09**	**0.78**	**4000.00**	0.60	−0.06	1.26	4000.00
*SS* vs. *LL* + *LS* × sex	–	–	–	–	–	–	–	–	−0.22	−0.93	0.59	3818.62
Extraversion												
Intercept	−0.19	−0.56	0.15	4000.00	−0.23	−0.60	0.14	4000.00	−0.44	−0.89	−0.02	4000.00
*SS* vs. *LL* + *LS*	0.25	−0.15	0.64	4000.00	0.22	−0.20	0.60	4000.00	**0.54**	**0.01**	**1.06**	**4000.00**
Sex	–	–	–	–	0.14	−0.20	0.49	4000.00	**0.68**	**0.02**	**1.34**	**4000.00**
*SS* vs. *LL* + *LS* × sex	–	–	–	–	–	–	–	–	−0.73	−1.48	0.05	4000.00
Conscientiousness												
Intercept	0.36	−0.00	0.69	3621.01	0.61	0.27	0.96	4189.74	0.65	0.27	1.08	4000.00
*SS* vs. *LL* + *LS*	**−0.44**	**−0.82**	**−0.03**	**4166.38**	−0.35	−0.73	0.01	4233.38	−0.41	−0.88	0.09	3739.51
Sex	–	–	–	–	**−0.71**	**−1.05**	**−0.39**	**3210.01**	**−0.83**	**−1.50**	**−0.19**	**3818.34**
*SS* vs. *LL* + *LS* × sex	–	–	–	–	–	–	–	–	0.16	−0.60	0.90	3791.20
Agreeableness												
Intercept	−0.15	−0.51	0.21	4000.00	−0.15	−0.50	0.23	4000.00	−0.16	−0.59	0.27	4000.00
*SS* vs. *LL* + *LS*	0.25	−0.15	0.66	4000.00	0.25	−0.14	0.66	4000.00	0.27	−0.24	0.78	4000.00
Sex	–	–	–	–	0.01	−0.35	0.35	4000.00	0.05	−0.64	0.75	3919.04
*SS* vs. *LL* + *LS* × sex	–	–	–	–	–	–	–	–	−0.06	−0.90	0.71	4000.00
Neuroticism												
Intercept	−0.13	−0.48	0.21	3795.22	−0.15	−0.53	0.21	4000.00	−0.25	−0.66	0.18	4000.00
*SS* vs. *LL* + *LS*	0.15	−0.27	0.55	4000.00	0.15	−0.23	0.58	4000.00	0.31	−0.20	0.82	4000.00
Sex	–	–	–	–	0.03	−0.30	0.40	4000.00	0.32	−0.37	1.01	3210.06
*SS* vs. *LL* + *LS* × sex	–	–	–	–	–	–	–	–	−0.39	−1.20	0.40	3070.15
Openness												
Intercept	−0.02	−0.38	0.31	4000.00	0.03	−0.33	0.41	4000.00	0.03	−0.41	0.45	4000.00
*SS* vs. *LL* + *LS*	0.03	−0.37	0.44	4000.00	0.05	−0.34	0.47	4000.00	0.05	−0.46	0.59	4000.00
Sex	–	–	–	–	−0.15	−0.51	0.20	4000.00	−0.15	−0.86	0.53	4000.00
*SS* vs. *LL* + *LS* × sex	–	–	–	–	–	–	–	–	0.01	−0.83	0.79	4000.00
Low alpha												
Intercept	−0.18	−0.54	0.16	4000.00	−0.40	−0.76	−0.04	4000.00	−0.47	−0.88	−0.03	4000.00
*SS* vs. *LL* + *LS*	0.20	−0.19	0.62	4000.00	0.13	−0.26	0.52	4146.43	0.24	−0.26	0.74	4354.32
Sex	–	–	–	–	**0.59**	**0.26**	**0.95**	**4000.00**	**0.78**	**0.11**	**1.41**	**3711.00**
*SS* vs. *LL* + *LS* × sex	–	–	–	–	–	–	–	–	−0.25	−1.01	0.50	4000.00
Disinhibition												
Intercept	−0.22	−0.57	0.13	4000.00	−0.41	−0.78	−0.05	4177.15	−0.49	−0.87	−0.05	4000.00
*SS* vs. *LL* + *LS*	0.26	−0.16	0.65	3693.09	0.20	−0.20	0.59	4369.26	0.32	−0.18	0.80	4000.00
Sex	–	–	–	–	**0.50**	**0.16**	**0.85**	**3742.39**	**0.73**	**0.09**	**1.37**	**4000.00**
*SS* vs. *LL* + *LS* × sex	–	–	–	–	–	–	–	–	−0.32	−1.04	0.45	4000.00
Negative emotionality/low dominance												
Intercept	0.03	−0.32	0.37	4000.00	0.21	−0.17	0.58	4000.00	0.22	−0.18	0.66	4000.00
*SS* vs. *LL* + *LS*	−0.05	−0.47	0.35	4000.00	0.00	−0.41	0.38	4000.00	−0.01	−0.50	0.50	4231.06
Sex	–	–	–	–	−0.46	−0.82	−0.12	4000.00	−0.50	−1.14	0.22	4000.00
*SS* vs. *LL* + *LS* × sex	–	–	–	–	–	–	–	–	0.05	−0.71	0.87	4000.00

Personality domains and hierarchical personality dimensions were converted into z-scores for these analyses. l-95 % and u-95 % represent the lower and upper bounds of the 95 % credible interval, respectively. N_eff_ = effective sample size. Significant values highlighted in bold (p_MCMC_ < 0.05)

### Supplementary analyses

Because the results of our linear models and our animal models differed from those of Hopkins et al. (2012) and Latzman et al. (2014), we used our data to test whether differences between our study and the earlier studies are responsible for these contrasting findings. The first difference was that our sample included 19 chimpanzees from Guinea who were orphaned early in life. The effect of being orphaned may have influenced the development of these chimpanzees either alone or via gene × environment interactions (Suomi 2006). So far as we are aware, the samples studied by Hopkins et al. (2012) and Latzman et al. (2014) did not include chimpanzees who were orphaned early in life, and they were all captive-housed. Another difference was that the chimpanzees in the present study were assessed using a 54 item questionnaire that defined six components. The chimpanzees in the Hopkins et al. (2012) study were assessed using the 43 item Chimpanzee Personality Questionnaire and the four domains examined in their study (Weiss et al. 2007) differed some from their counterparts in our study.

To test whether these differences explain why we did not find the same results as did Hopkins et al. (2012) and Latzman et al. (2014), we fit animal models that included sex, genotype, and sex × genotype as fixed effects, and subject ID conditioned by relatedness as a random effect. Our priors, covariance matrix, number of iterations, burn in period, and thinning were identical to our main analyses. However, for these animal models, we excluded the 19 chimpanzees from Guinea and used unit-weighted scores for dominance and conscientiousness that were identical to those described by Hopkins et al. (2012) (see ESM Table S1). Trace plots did not suggest the presence of autocorrelations and density plots indicated that the distributions around the estimates were approximately normal. Again, data used to create these plots are available at https://github.com/alexweissuk/avpr1a-chimpanzee.git. The analyses indicated that males were significantly higher than females in low alpha; none of the other fixed effects of sex nor the effects of genotype and none of the sex × genotype interaction were significant (see Table 4).

**Table 4:** MCMCglmm results for the effects of AVPR1 genotype, sex, and sex × genotype on personality domains and hierarchical personality dimensions when personality domains were defined as they were in Weiss et al. (2007) and chimpanzees living in Guinea are excluded

	b	l-95 %	u-95 %	N_eff_
Dominance				
Intercept	−0.30	−0.75	0.16	4000.00
*SS* vs. *LL* + *LS*	0.05	−0.48	0.60	4000.00
Sex	0.70	−0.05	1.45	3812.24
*SS* vs. *LL* + *LS* × sex	−0.15	−0.96	0.77	4000.00
Conscientiousness				
Intercept	0.55	0.10	1.00	4000.00
*SS* vs. *LL* + *LS*	−0.46	−1.01	0.10	4000.00
Sex	−0.62	−1.38	0.15	4000.00
*SS* vs. *LL* + *LS* × sex	0.22	−0.65	1.04	4000.00
Low alpha				
Intercept	−0.47	−0.93	−0.03	4000.00
*SS* vs. *LL* + *LS*	0.31	−0.27	0.83	3822.28
Sex	**0.77**	**0.08**	**1.56**	**4287.68**
*SS* vs. *LL* + *LS* × sex	−0.30	−1.12	0.54	4090.68
Disinhibition				
Intercept	−0.47	−0.90	−0.00	4000.00
*SS* vs. *LL* + *LS*	0.41	−0.11	0.99	4000.00
Sex	0.65	−0.07	1.43	3578.12
*SS* vs. *LL* + *LS* × sex	−0.33	−1.18	0.54	3419.31
Negative emotionality/low dominance				
Intercept	0.21	−0.24	0.65	3945.26
*SS* vs. *LL* + *LS*	0.03	−0.49	0.58	4105.81
Sex	−0.62	−1.35	0.13	4000.00
*SS* vs. *LL* + *LS* × sex	0.12	−0.72	0.98	3875.40

Personality domains and hierarchical personality dimensions were converted into z-scores for these analyses. l-95 % and u-95 % represent the lower and upper bounds of the 95 % credible interval, respectively. N_eff_ = effective sample size. Significant values highlighted in bold (p_MCMC_ < 0.05)

One further possibility is that differences in the distribution of chimpanzees and raters across facilities led to our results. Specifically, in contrast to Hopkins et al. (2012) and Latzman et al. (2014), where all of the chimpanzees belonged to a single facility and their personalities were assessed by a largely overlapping set of raters, although the majority of our captive sample (n = 77) were housed in a single sanctuary and had their personalities assessed by one group of raters, 33 chimpanzees were housed across ten institutions, each with a different set of raters. To test whether this explained our results we fit a linear model and animal model that included the effects of sex, genotype, and sex × genotype in the chimpanzees who lived in the sanctuary. For these analyses we focused on the version of the conscientiousness domain examined by Hopkins et al. (2012). The linear model revealed that males were rated as significantly lower in conscientiousness than females (b = −0.91, 95 % CI −1.80 to −0.02, p = 0.044), that chimpanzees homozygous for the short form of the allele were significantly lower in conscientiousness than chimpanzees who possessed the long form (b = −0.73, 95 % CI −1.37 to −0.08, p = 0.028), and that there was no significant sex × genotype interaction (b = 0.76, 95 % CI −0.27 to 1.79, p = 0.14). Trace plots for the animal model did not suggest the presence of autocorrelations and density plots indicated that the distributions around the estimates were approximately normal. Again, the data used to create these plots is available at https://github.com/alexweissuk/avpr1a-chimpanzee.git. The animal model results were similar: chimpanzees who had lower conscientiousness scores were male (b = −1.06, 95 % CI −1.96 to −0.13, n_eff_ = 3898, p_MCMC_ = 0.026) and possessed the *SS* genotype (b = −0.71, 95 % CI −1.43 to −0.08, n_eff_ = 4000, p_MCMC_ = 0.043). Once again, interaction was not significant (b = 0.93, 95 % CI −0.10 to 1.98, n_eff_ = 4000, p_MCMC_ = 0.0860).

## Discussion

We found an association between higher conscientiousness and the long form of the AVPR1a gene in a linear model and in an animal model. In both cases, the effect of genotype was not significant when adjusting for sex or for sex and sex × genotype, although supplementary analyses suggested that this may be attributable to the way that the chimpanzees and raters in our study were distributed across facilities. We also found that *S* homozygotes were higher in extraversion. We found no evidence for associations between AVPR1a genotype and the dominance, agreeableness, neuroticism, and openness domains or the hierarchical personality dimensions. The credible intervals for the narrow sense heritability estimates of all six personality domains and the three hierarchical personality dimensions did not include zero.

Previous studies of chimpanzees found that the long form of AVPR1a was associated with dominance and conscientiousness (Hopkins et al. 2012), and hierarchical personality dimensions related that captured conscientiousness- and dominance-related traits (Latzman et al. 2014), but that the direction of these effects differed between males and females. Other findings in chimpanzees found associations between the long form of the gene and better performance in a joint attention task (Hopkins et al. 2014), a tendency to use coalitions and receive positive attention from conspecifics and, among males, to have many friends (Anestis et al. 2014), and a higher frequency of allogrooming (Staes et al. 2015). Studies of humans found an association between the AVPR1a genotype and aggression in children similar to what has been found in other species (Pappa et al. 2016), and an association between the long allele of the RS3 region and altruistic behavior, and increased expression of AVPR1a mRNA in the hippocampus (Knafo et al. 2008).

A study of captive chimpanzees found an association between conscientiousness and lower levels of agonistic behavior (Pederson et al. 2005) and a content analysis of conscientiousness revealed one facet related to predictability and low impulsivity and another related to low levels of aggression (King and Weiss 2011). These findings, then, along with the above-described studies of chimpanzees and humans, suggest that the long form of the AVPR1a gene acts to reduce levels of impulsive aggression. The long form of the AVPR1a gene, then, might reduce impulsive aggression by promoting social perception (Hopkins et al. 2014) and/or promoting socially appropriate behavior (Anestis et al. 2014; Bachner-Melman et al. 2005; Staes et al. 2015).

On the other hand, some of our findings do not mesh well with what we would expect based on previous findings. For one, our finding that *L* carriers were lower in extraversion is the opposite of what would be expected from a gene that is related to social behaviors and also findings that chimpanzees who possess the long form of AVPR1a exhibit higher levels of allogrooming (Staes et al. 2015). One possibility is that the traits captured by chimpanzee (Freeman and Gosling 2010; King and Figueredo 1997; Weiss et al. 2007, 2009) and human extraversion (Costa and McCrae 1995), for example, gregariousness, activity, and positive affect, have a different and opposite association with the long form of AVPR1a than do traits related to socially appropriate behaviors, such as a tendency to avoid unnecessary aggression. However, because this association was only significant in an animal model that adjusted for sex and sex × genotype and its effect size was larger in these models than in the unadjusted and the sex-adjusted models, we would advise caution in interpreting this result until it can be examined in further studies or in a meta-analysis.

Another puzzling finding was that unlike Hopkins et al. (2012) and Latzman et al. (2014) we did not find evidence for sex × genotype effects for conscientiousness, dominance, or the hierarchical personality dimensions. Results from our first supplementary analyses suggested that these null results were not attributable to the dominance and conscientiousness scales used or our inclusion of a group of wild, orphaned chimpanzees. In addition, our second supplementary analyses could not clearly rule out (or in) the possibility that the way in which chimpanzees and raters were distributed across facilities in our study led to our null findings with respect to whether the effect of genotype on conscientiousness differed between males and females. Of course, one remaining possibility to explain our discrepant findings is that we used animal models to control for relatedness whereas Hopkins et al. (2012) and (Latzman et al. 2014) did not. However, we do not think this is likely given that Hopkins et al. (2014) found a significant sex by AVPR1a genotype interaction using an analysis method that controlled for relatedness in much the same way as does the animal model (Almasy and Blangero 1998). Given these findings, we do not think that methodological differences between our study and those of Hopkins et al. (2012) and Latzman et al. (2014) can explain our somewhat different results. Further research on larger samples, preferably assessed by the same group of raters, is thus needed to resolve this question.

In addition to the findings related to the AVPR1a genotype and personality domains and hierarchical personality dimensions, the evidence for the heritability of all of the personality phenotypes is consistent with studies of humans (Bouchard and Loehlin 2001), orangutans (Adams et al. 2012), rhesus macaques (Brent et al. 2014), and chimpanzees (Latzman et al. 2015; Weiss et al. 2000). Weiss et al. (2000) reported a heritability of 0.63 for chimpanzee dominance, an estimate higher than that reported here. The same study also found no evidence for the heritability of the other factors. One possible reason for these differences may be that the prior study used symmetric differences squared, which relies on ordinary least squares regression (Grimes and Harvey 1980). In contrast, the present study implemented the animal model using Bayesian analysis, which performs better when sample sizes are relatively small (O’Hara et al. 2008).

Trying to understand the genetic basis of complex traits has given rise to debate over the best approach to assessing personality-genotype associations. Some argue that genome-wide association studies are preferable to candidate gene studies because they account for the fact that complex traits may be influenced by small effects of multiple genes (Chabris et al. 2012). However, a genome wide association study of chimpanzees or any other great ape species is not feasible as obtaining sufficient sample sizes for such studies would be impossible. Furthermore, candidate gene studies are beneficial if they are hypothesis-driven and selection of the candidate gene is based on knowledge of the functional role of the polymorphism (Tabor et al. 2002). Thus candidate gene studies, including attempts to replicate findings, may complement genome-wide association studies (Reif and Lesch 2003).

Understanding differences in the association between AVPR1a and social behavior across species has important consequences for how we understand the evolution of group cohesion and cooperation. High powered studies testing for associations between AVPR1a and personality measures that are standardized across species would be beneficial to this end.

## Electronic supplementary material

Below is the link to the electronic supplementary material.

Supplementary material 1 (DOCX 24 kb)
