# Supplementary material for: Chimpanzee Personality and the Arginine Vasopressin Receptor 1A Genotype
Source: Behav Genet. 2016 Nov 2;47(2):215–26. doi: 10.1007/s10519-016-9822-2 (PMC5306277; doi:10.1007/s10519-016-9822-2)
Supplement: Supplementary file 1 — Supplementary material 1 (DOCX 24 kb) [file 10519_2016_9822_MOESM1_ESM.docx]

**Electronic Supplementary Material Table 1:** Unit weighted items for each chimpanzee personality domain, for the six factor structure ([Weiss et al. 2009](#_ENREF_50)), four factor structure ([Hopkins et al. 2012](#_ENREF_26)) and the three hierarchical personality dimensions ([Latzman et al. 2014](#_ENREF_34))

|  | Six factor structure | | Four item structure | |
| --- | --- | --- | --- | --- |
|  | Loading | | Loading | |
| Domain^a^ | Positive | Negative | Positive | Negative |
| Dominance | Dominant  Independent  Decisive  Intelligent  Persistent  Bullying  Stingy  Manipulative | Submissive  Dependent  Fearful  Timid  Cautious  Vulnerable  Anxious | Dominant  Independent  Decisive  Persistent  Bullying | Submissive  Dependent  Fearful  Timid  Cautious |
| Extraversion | Active  Playful  Social  Friendly  Affectionate  Imitative | Solitary  Lazy  Individualistic  Depressed | Active  Playful  Sociable  Friendly  Affectionate  Inquisitive  Inventive  Imitative | Solitary  Lazy  Depressed  Unemotional |
| Conscientiousness | Predictable | Impulsive  Defiant  Reckless  Erratic  Irritable  Aggressive  Jealous  Disorganized  Thoughtless  Distractible  Unperceptive  Quitting  Clumsy | Predictable  Stable  Gentle | Impulsive  Defiant  Reckless  Erratic  Irritable  Aggressive  Jealous  Disorganized  Clumsy  Depressed  Excitable  Autistic  Stingy  Manipulative |
| Agreeableness | Sympathetic  Helpful  Sensitive  Protective  Gentle  Conventional |  | Sympathetic  Helpful  Sensitive  Protective  Gentle  Sociable  Friendly  Affectionate  Stable  Predictable  Intelligent |  |
| Neuroticism | Excitable  Autistic | Stable  Cool |  |  |
| Openness | Inquisitive  Inventive  Curious  Innovative |  |  |  |
| Hierarchical dimension^b^ | Positive | | Negative | |
| Disinhibition | Impulsive  Erratic  Reckless  Excitable  Irritable  Aggressive  Bullying  Defiant  Jealous  Stingy | | Stable  Gentle  Sympathetic  Friendly  Predictable  Affectionate | |
| Negative Emotionality | Dependent  Submissive  Timid  Fearful  Cautious | | Bullying  Independent  Dominant  Decisive | |
| Low Alpha | Aggressive  Bullying  Reckless  Defiant  Dominant  Stingy  Irritable  Impulsive  Jealous  Persistent  Erratic  Excitable  Independent | | Gentle  Cautious  Submissive  Sympathetic  Dependent  Stable  Friendly | |

Note. ^a^Item weights generated from loadings ≥ |0.4|; ^b^Item weights generated from loadings ≥ |0.5| as in [Latzman et al. (2014)](#_ENREF_34).
